# Predictive impact of rare genomic copy number variations in siblings of individuals with autism spectrum disorders

**DOI:** 10.1038/s41467-019-13380-2

**Published:** 2019-12-05

**Authors:** L. D’Abate, S. Walker, R. K. C. Yuen, K. Tammimies, J. A. Buchanan, R. W. Davies, B. Thiruvahindrapuram, J. Wei, J. Brian, S. E. Bryson, K. Dobkins, J. Howe, R. Landa, J. Leef, D. Messinger, S. Ozonoff, I. M. Smith, W. L. Stone, Z. E. Warren, G. Young, L. Zwaigenbaum, S. W. Scherer

**Affiliations:** 1 The Centre for Applied Genomics, Genetics, and Genome Biology, The Hospital for Sick Children, Toronto, ON Canada; 2 Department of Molecular Genetics, University of Toronto, Toronto, ON Canada; 3 Center of Neurodevelopmental Disorders at Karolinska Institutet (KIND), Department of Women’s and Children’s Health, Stockholm, Sweden; 4 Center for Psychiatry Research, Region Stockholm, Stockholm, Sweden; 5 Autism Research Centre, Bloorview Research Institute and University of Toronto, Toronto, ON Canada; 6 Autism Research Centre, IWK Health Centre and Dalhousie University, Halifax, NS Canada; 7 Department of Psychology, UC San Diego, La Jolla, CA USA; 8 Center for Autism and Related Disorders, Kennedy Krieger Institute, Baltimore, MD USA; 9 Department of Psychology, University of Miami, Coral Gables, FL USA; 10 MIND Institute, Department of Psychiatry, UC Davis, Davis, CA USA; 11 Department of Psychology, University of Washington, Seattle, WA USA; 12 Vanderbilt Kennedy Center Treatment and Research Institute for Autism Spectrum Disorders, Vanderbilt Kennedy Centre, Nashville, TN USA; 13 Autism Research Centre, University of Alberta, Edmonton, AB Canada; 14 McLaughlin Centre, University of Toronto, Toronto, ON Canada

**Keywords:** Rare variants, Structural variation, Predictive markers, Autism spectrum disorders

## Abstract

Identification of genetic biomarkers associated with autism spectrum disorders (ASDs) could improve recurrence prediction for families with a child with ASD. Here, we describe clinical microarray findings for 253 longitudinally phenotyped ASD families from the Baby Siblings Research Consortium (BSRC), encompassing 288 infant siblings. By age 3, 103 siblings (35.8%) were diagnosed with ASD and 54 (18.8%) were developing atypically. Thirteen siblings have copy number variants (CNVs) involving ASD-relevant genes: 6 with ASD, 5 atypically developing, and 2 typically developing. Within these families, an ASD-related CNV in a sibling has a positive predictive value (PPV) for ASD or atypical development of 0.83; the Simons Simplex Collection of ASD families shows similar PPVs. Polygenic risk analyses suggest that common genetic variants may also contribute to ASD. CNV findings would have been pre-symptomatically predictive of ASD or atypical development in 11 (7%) of the 157 BSRC siblings who were eventually diagnosed clinically.

## Abstract (web summary)

Siblings of those with autism spectrum disorder (ASD) have increased likelihood of ASD or related subclinical traits. Here, studying 253 ASD families, D’Abate et al. test the predictive value of genomic copy number variation involving ASD-associated loci, with confirmation in a second cohort.

## Introduction

Behavioral assessments remain the gold standard for autism spectrum disorder (ASD) diagnosis^1^, and prospective analysis permits objective and longitudinal assessment for the earliest symptoms. Published estimates of sibling recurrence for ASD range from 6.9 to 19.5%^2–5^. Moreover, of younger siblings of autistic probands, herein referred to simply as “infant siblings”, who are not diagnosed with ASD, up to 30–40% have subclinical ASD traits and/or suboptimal developmental functioning^6^.

ASD and related subclinical traits show familial clustering, with a substantial portion of familial liability attributed to genetic factors^7,8^. Subclinical symptoms in first- and second-degree relatives support an important role for genetic factors in producing an autistic phenotype^9^. The genetic architecture of ASD is being resolved by studying families with different characteristics^10–21^, and dozens of copy number variant (CNV) loci and ASD-relevant genes and loci are known, many of which overlap those associated with other neurodevelopmental disorders^14,17,18^. De novo and inherited rare (<1% in a population) CNVs and other pathogenic variants are found in ~ 5–40% of individuals with ASD, depending on the cohort examined^10,14,17,20^. Chromosomal microarray to detect CNVs is the first-tier laboratory test for clinical genetic evaluation following an ASD diagnosis^22^.

**Fig. 1:**
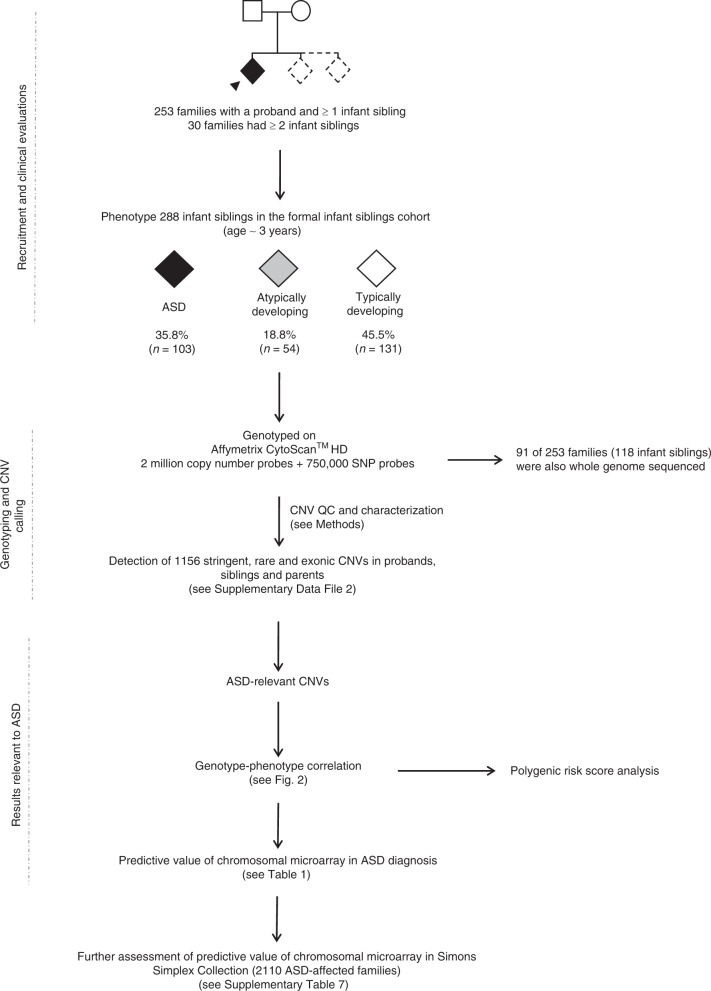
Project flowchart. Families consisting of a proband and at least 1 infant sibling were recruited through the Baby Siblings Research Consortium. The proband was the first in the family to receive an ASD diagnosis. Psychometric data were collected for all siblings at ~36 months of age, at which point an ASD diagnosis was made if the child met clinical criteria (see Methods). All children were also assessed for ASD, cognitive and adaptive behavioral functioning at least once prior to the 36-month time point. We genotyped individuals from 253 families on the Affymetrix CytoScan^TM^ HD Array and whole-genome sequenced 91 of these families using established pipelines^12–14^. Copy number variants determined to be ASD-relevant were confirmed with secondary methods. We scrutinized the phenotypes of high-risk infant siblings carrying these ASD-relevant CNVs to determine whether they (i) had ASD, (ii) were atypically developing, or (iii) were neurotypical/non-ASD. There were also 34 siblings who did not meet criteria for formal enrollment but for whom phenotype and microarray data were available. Following the general BSRC strategy, a separate CNV analysis was conducted on 2124 probands and 2423 non-ASD sibs from 2110 ASD-affected families part of the Simons Simplex Collection. Fourteen probands were monozygotic twins. Of the unaffected sibs, 288 were atypically developing. This analysis was performed to further assess the predictive value of chromosomal microarray. ASD autism spectrum disorder, CNV copy number variation.

The most effective way to reduce symptoms of ASD is with early intervention, targeting behavior, and skills development^23^. In search of biomarkers for early indentification, we investigated whether CNVs affecting ASD-related loci correlate (pre- and post-symptomatically) with phenotypic outcomes in the Baby Siblings Research Consortium (BSRC) cohort of infant siblings whose family history is associated with a higher probability of developing ASD (Fig. 1). We analyze CNVs from 253 families registered in the BSRC^24^ while blinded to the infant siblings’ phenotype status. At enrollment, each family included a proband diagnosed with ASD, and at least 1 younger sibling (Supplementary Table 1). The BSRC longitudinal phenotyping design enables a predictive study of CNVs in this infant cohort (see Methods). Our analyses reveal that the detection of ASD-relevant CNVs is indeed predictive of ASD or atypical development in this sibling population and can be used to inform risk estimates for individuals and their families, with potential impact on their therapeutic trajectory.

## Results

### Clinical evaluation of infant siblings within families

The study group included the 253 probands, 288 later-born infant siblings comprising the primary diagnostic sample (Fig. 1), and 34 siblings who did not meet cohort enrollment criteria (i.e., >3 years, or status determined only through parental reports), but for whom we had phenotype designation and genotype data. Refer to “Subject recruitment and clinical evaluations” in Methods section for criteria for various atypical outcomes listed below. At age 3, 103/288 siblings (35.8%) in 94/253 families (37.2%) had a formal diagnosis of ASD; 54 were “atypically developing” (18.8%), and 131 were developing typically (45.5%) (Supplementary Table 2). Among the 54 infant siblings with atypical development (but not an ASD diagnosis) were the following phenotypic constellations: ASD symptoms (*n* = 5), ASD symptoms with developmental, language and/or adaptive behavior delay (*n* = 9); developmental delay (*n* = 15), language delay (*n* = 10), language and adaptive behavior delays (*n* = 5), deficits in adaptive behavior alone (*n* = 3), and attention deficit hyperactivity disorder (ADHD) symptoms or externalizing behaviors (*n* = 7). Of the 30 families with 2 or more infant siblings, 23 had 1 or more assessed as having ASD and/or atypical development and 7 had all sibs typically developing at age 3. The male:female ratio was 3.7:1 for ASD-affected infant siblings, 1.6:1 for atypically developing siblings, and 1.2:1 for non-ASD infant siblings.

### Genomic findings in probands and infant siblings

**Fig. 2:**
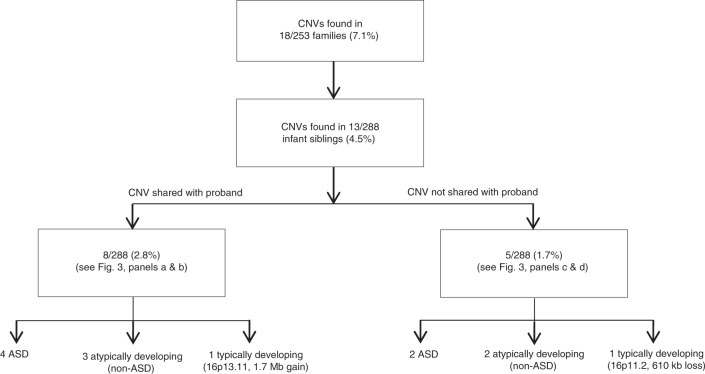
ASD-relevant CNVs among infant siblings (*n* = 288). Summary of ASD-relevant genetic findings in the infant sibling cohort, stratified by family segregation and infant sibling phenotype. ASD autism spectrum disorder, CNV copy number variation.

**Fig. 3:**
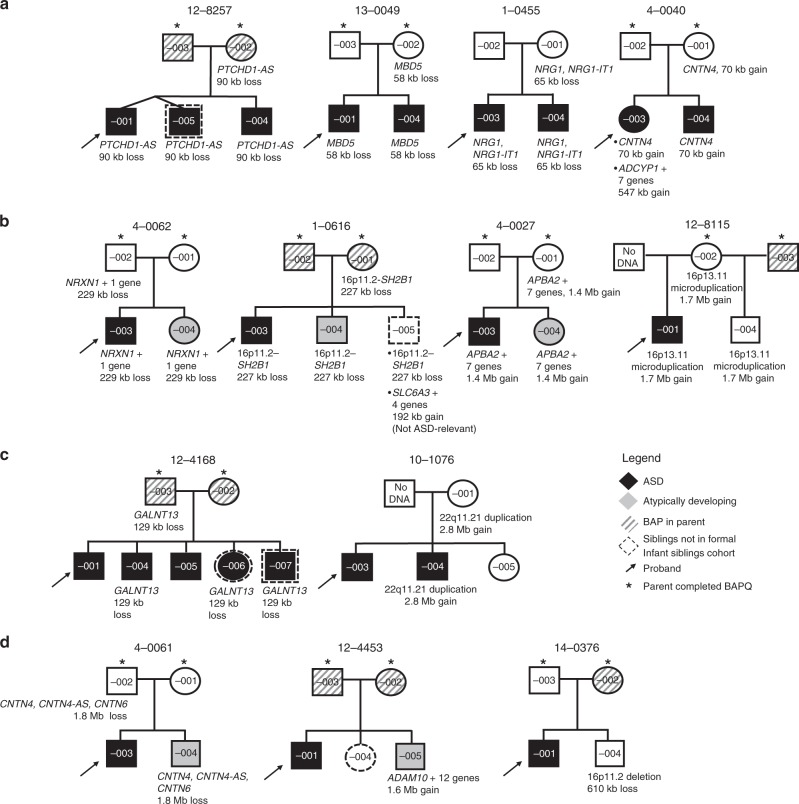
Pedigrees demonstrating ASD-relevant CNVs in infant siblings. **a** Infant siblings with ASD who shared a CNV with a related index case (arrow). Targeted testing (only) for a pathogenic CNV was performed on sibling 12-8257-005. **b** Atypically developing infant siblings who shared a CNV with a related index case. Female sibling 4-0062-004 was positive for ASD on the ADOS (24 and 36 months) assessments, but subthreshold on the ADI-R. Clinical impression was that the child did not have ASD. She had subthreshold scores on the MSEL Early Learning Composite (ELC) (SS = 76) and VABS Adaptive Behavior Composite (ABC) (SS = 84). In family 1-0616, the second-born son (1-0616-004) displayed subthreshold ASD symptoms, vulnerabilities in language and verbal reasoning, gross and fine-motor skill delay; the third-born son (1-616-005) (not in the official cohort) experienced fine-motor delay as seen on the VABS at 24 months, and parents reported concerns regarding socialization. In family 4-0027, the female sibling displayed behavioral rigidity and transition difficulties. The male sibling in family 12-8115 was developing typically. **c** Infant siblings with ASD who did not share a CNV with a related index case. **d** Infant siblings with a CNV not shared with a related index case. Individual 4-0061-004 had language delay and cognitive regression (ELC = 56). Male sibling 12-4453-005 scored just above threshold on the ADOS (CSS = 5), but the clinical history was not consistent with a diagnosis of ASD. His ELC (SS = 88), and ABC (SS = 90) scores were within 1 standard deviation of the expected mean. The scores of male 14-0376-001 on the ADOS (CSS = 1), MSEL (ELC = 91) and the VABS (ABC = 105) reflected a typical developmental trajectory. Figure includes 5 additional non-infant siblings who were not counted as part of the formal cohort (dotted outline). CNV classification is provided in Supplementary Table 4. ASD autism spectrum disorder, CNV copy number variation, ADOS Autism Diagnostic Observation Schedule, CSS calibrated severity score, ADI-R Autism Diagnostic Interview-Revised, MSEL Mullen Scales of Early Learning, VABS Vineland Adaptive Behavior Scales, SS standard score.

From 253 probands, we identified 15 CNVs (in 13 individuals; 5.1%) that were deemed ASD-relevant (see Methods; Supplementary Tables 3 and 4). Where inheritance could be ascertained, 6 CNVs (in 5 probands) were de novo (3 deletions, 3 duplications) and 8 CNVs (5 deletions, 3 duplications) were inherited (7 maternally, 1 paternally). For proband 14-0152-001, inheritance was unknown, as parental samples were unavailable. All 8 inherited variants were shared with an infant sibling (Fig. 2), of whom 4 were diagnosed with ASD (Fig. 3a) and 3 showed atypical development without a formal ASD diagnosis (Fig. 3b).

Among the 288 infant siblings of the probands, 13 carried ASD-relevant CNVs (Fig. 2), of which 6 were considered pathogenic and 7 were clinically defined as variants of unknown significance overlapping genes implicated in ASD. Five infant siblings had ASD-relevant CNVs that were not shared with the related probands; 2 had ASD, 2 were atypically developing, and 1 was typically developing (Fig. 3c, d). Of the 2 typically developing children with ASD-relevant CNVs, 14-0376-004 (Fig. 3d) had a 610 kb deletion at 16p11.2 and 12-8115-004 (Fig. 3b) had a (shared) 1.7 Mb duplication at 16p13.11. Their evaluations at age 36 months did not indicate any developmental delays or ASD symptoms. Both variants are associated with recurrent and variably expressed syndromes, often involving ASD^12,25,26^.

Overall, among siblings of ASD probands, we found ASD-related CNVs in 6 of 103 with ASD at age 3 years, in 5 of 54 with atypical development, and in 2 of 131 with neurotypical development. Four children (Fig. 3) who did not meet study inclusion criteria had ASD-related CNVs deemed to be pathogenic. Using only CNVs considered to be pathogenic or likely pathogenic^18,27^, among infant siblings the positive predictive values (PPVs) of these variants were 0.50 for ASD and 0.83 for combined ASD/atypical development. Other predictive statistics are shown in Table 1.

**Table 1:** Predictive statistics of microarray findings in infant siblings of ASD probands.

	ASD (all ASD-relevant CNVs)	ASD or atypical development (all ASD-relevant CNVs)	ASD (excluding VUS)	ASD or atypical development (excluding VUS)
Statistic				
Sensitivity (0.95 CI)	0.06 (0.02–0.12)	0.07 (0.04–0.12)	0.03 (0.01–0.08)	0.03 (0.01–0.07)
Specificity (0.95 CI)	0.96 (0.92–0.98)	0.98 (0.95–1.00)	0.98 (0.95–1.00)	0.99 (0.96–1.00)
Positive predictive value (0.95 CI)	0.46 (0.19–0.75)	0.85 (0.55–0.98)	0.50 (0.12–0.88)	0.83 (0.36–1.00)
Negative predictive value (0.95 CI)	0.65 (0.59–0.70)	0.47 (0.41–0.53)	0.65 (0.59–0.70)	0.46 (0.40–0.52)
Genotype/phenotype				
Condition +	103^a^	157^b^	103^a^	157^b^
CNV +/condition +	6	11	3	5
CNV +/condition –	7	2	3	1
CNV –/condition –	178	129	182	130
CNV –/condition +	97	146	100	152
Total infant siblings	288	288	288	288

*VUS* variant of unknown significance

Genotype refers to the identification of ASD-relevant CNVs observed in infant siblings

^a^Affected sibs (*n* = 103)

^b^Affected sibs (*n* = 103) + non-ASD sibs with atypical development (*n* = 54) (see “Subject recruitment and clinical evaluations” in Methods)

Whole-genome sequences (WGS) were available from 91 families of our cohort. We identified no additional ASD-relevant CNVs, and of the potentially interesting sequence-level variants (Supplementary Table 5), none were found in families with previously recognized ASD-relevant CNVs (Supplementary Fig. 1).

This study adds to growing evidence that specific biomarkers might contribute to pre-symptomatic detection of infants likely to develop ASD or other developmental disorders, at least from sibships that include an ASD proband. Eleven of 157 siblings who developed ASD or were atypically developing at age 3 carried a CNV worthy of clinical follow-up (either inherited or de novo), 5 (3.2%) carried a pathogenic/likely pathogenic CNV and 6 (3.8%) carried a variant of unknown significance (VUS).

Of the 7 total VUSs overlapping a gene associated with a neuropsychiatric disorder, 6 were in siblings with ASD or atypical development, with none in neurotypical children, despite nearly equal numbers in each subgroup. Although not reported as pathogenic in a clinical setting, VUSs involving ASD-relevant genes may nonetheless be contributing factors for ASD or atypical development, and of interest to families^14,22^. Two of 131 typically developing siblings had CNVs considered as VUS or likely pathogenic. These CNVs at 16p11.2 or 16p13.11 have frequencies in the general population that range from 0.03 to 0.04% and 0.15 to 0.25%^28–30^ and have been associated with reduced cognitive and general functioning^28,31^ and adult-onset phenotypes^32^, respectively.

To further assess the predictive impact of ASD-relevant CNVs in a larger cohort, we analyzed published data from 2110 families from the Simons Simplex Collection (SSC). This cohort differs from the BRSC in being a quartet design with 4214 parents, 2124 ASD-affected probands and 2423 ASD-unaffected children, assembled to study de novo variants (single-point phenotyping; average age of diagnosis 8.9 (±3.5) years for males and 9.11 (±3.7) for females; 6.6:1 male to female ratio for probands)^33^. Of the SSC “unaffected” sibs, applying similar criteria as for the BSRC cohort revealed 288 (11.9%) with atypical behavioral and developmental profiles: 33 were suspected to have elevated ASD traits (Social Responsiveness Scale-Parent Report Total *T*-Score > 60^34^); 139 had emotional and behavioral problems (Child Behavioral Checklist-Parent Report Total Problems *T*-Score > 60^35^); 65 displayed mild-moderate adaptive behavior deficits (Vineland Adaptive Behavior Scales, Adaptive Behavior Composite^36^ < 85); 51 met criteria in more than 1 category. While blinded to proband and sibling status, we searched for ASD-relevant CNVs with the same classification criteria used for the BSRC (see Methods).

We found 118 ASD-relevant CNVs in 116 of 2124 probands (5.5%; 72.0% (85) pathogenic/likely pathogenic and 28.0% (33) VUS) and 64 CNVs in 63 of 2423 unaffected sibs (2.6%; 34.4% (22) pathogenic/likely pathogenic and 65.6% (42) VUS) (Supplementary Data File 1 and Supplementary Table 6). These results are similar to those found in probands and unaffected sibs in the BSRC cohort (5.1% and 3.8%, respectively). Seven (11.1%) of these sibs with ASD-relevant CNVs were deemed atypically developing. When considering pathogenic and likely pathogenic variants across the SSC, the PPV for ASD was 0.79, and for ASD or atypical development was 0.83 (Supplementary Table 7). From the SSC cohort, we identified 47 CNVs impacting 8 of the genes/loci harboring ASD-relevant CNVs in the siblings of the BSRC. Thirty of these CNVs were carried by probands, 2 by atypically developing sibs and 15 by unaffected sibs (Supplementary Table 6). The percentage of ASD-relevant CNVs in unaffected sibs in the SSC is similar to that observed in unaffected sibs in the BSRC. Furthermore, data analyzed from both cohorts yielded lower percentages of ASD-relevant variants in non-ASD sibs compared with probands, while noting that select variants are found in symptomatic carriers in the general population.

### Polygenic transmission disequilibrium test

Recognizing emerging evidence for the role of combinations of common genetic variants in ASD susceptibility^13^ and their potential efficacy in predicting ASD risk, we determined the contribution of polygenic risk scores (PRS) to the phenotype in the BSRC cohort using the polygenic transmission disequilibrium test^13^. In families for which genotype data were available for both parents, probands and at least 1 infant sibling, we observed a statistically significant over-transmission of risk variants from parents to probands (*n* = 189, mean difference (cohort *z*-score based) = 0.13, *p* = 0.01). There were non-significant differences of risk transmission for unaffected siblings (*n* = 112, mean = −0.003, *p* = 0.97), atypically developing siblings (*n* = 44, mean = −0.004, *p* = 0.97) and ASD-affected siblings (*n* = 93, mean = 0.07, *p* = 0.27) (Supplementary Fig. 2). Power to reject the null hypothesis (assuming the PRS explained 2.45% of phenotypic variance^37^) was ~98% for the proband (*n* = 189) and 56% for the affected sibling (*n* = 93). The significant over-transmission of risk from parents to probands suggested that common genetic variants may contribute to the phenotype in the BSRC sample. This PRS analysis in the SSC revealed a similar statistical trend. Using a paired Student’s *t-*test, we observed a significant difference in risk of transmission for SSC probands (*n* = 2118, mean = 0.547, *p* < 1e-10) and a non-significant risk of transmission for unaffected sibs (*n* = 2130, mean = −0.021, *p* = 0.116) and atypically developing sibs (*n* = 286, mean = −0.012, *p* = 0.744).

## Discussion

Early identification of the CNVs described in this study could be used to tailor recurrence risk estimates to individual families, with potential for intensified surveillance for infants at increased likelihood due to positive CNV findings. There is evidence that infants as young as 7 months with subtle features of ASD could benefit from tailored interventions^23,38,39^, but there are also also recent negative trial data^40,41^.

This study had possible limitations. First, given the 37% recurrence of ASD in our BSRC families, compared with 6.9–19.5% found previously^2–5^, this cohort had selective over-recruitment of infant siblings with ASD. A similar over-recruitment was noted for atypically developing sibs, which partially accounts for the difference in rate between cohorts (29.2% in BSRC compared to 11.9% in SSC). This oversampling, not uncommon in genetic studies, may bias estimates of positive and negative predictive value of biomarkers. As well, PPV estimates related to CNV detection must be considered relative to overall ASD rates among participants. Further impacting the predictive value statistics, we only considered diagnoses made by 3 years of age. Despite the stability of an ASD diagnosis at 36 months, clinical phenotypes are known to be fluid throughout development and apparent non-ASD siblings might later demonstrate ASD or another disorder as they age^42,43^. Likewise, longitudinal studies following high-probability siblings from 3 years of age to middle childhood observed that an ASD diagnosis was revoked in 5.6–20% of children^44,45^. Second, considering the massive effort in phenotyping, the BSRC sample would be considered comparably large, but participants with ASD-relevant CNVs were still few, limiting power of the analysis. The primary BSRC study involved a cohort with increased probability of ASD, and while the findings are generally consistent with those from the SSC, other family structures may yield different findings.

The recurrent CNVs described here often display variable expressivity and reduced penetrance for ASD, particularly when sex is considered^46^. For these reasons, we explored inclusion of siblings with atypical forms of ASD when determining PPV. We attribute the low sensitivity (0.03 in BSRC) of these markers to the vast heterogeneity in ASD etiology, but this parameter may improve with clearer genotype-phenotype correlations^17,47^. We can also consider coupling these already useful CNV indicators of ASD with metrics captured by other genetic approaches such as WGS and PRS discussed here, as well as other techniques, such as brain imaging^48^ and other early phenotypic assessment^49^.

Families must receive adequate counseling when such variants are found, at any stage of life, including the prenatal setting, so that they fully understand the potential ramifications of these findings. This sibling cohort also provided evidence for certain CNVs as potential early biological predictors of ASD or other developmental difficulties, whether or not they shared these with their respective probands (Fig. 2), which will also influence interpretation.

## Methods

### Subject recruitment and clinical evaluations

From 9 research sites registered with the BSRC (The Center for Applied Genomics, Genetics, and Genome Biology, The Hospital for Sick Children; Autism Research Center, Bloorview Research Institute; Autism Research Center, IWK Health Center and Dalhousie University; Department of Psychology, UC San Diego; Center for Autism and Related Disorders, Kennedy Krieger Institute; Department of Psychology, University of Miami; MIND Institute, Department of Psychiatry, UC Davis; Department of Psychology, University of Washington; Vanderbilt Kennedy Center Treatment and Research Institute for Autism Spectrum Disorders, Vanderbilt Kennedy Center; Autism Research Center, University of Alberta), participants from 253 families enrolled (134 from USA sites and 119 from Canada). Recruitment was through organizations serving individuals with ASD and their families, referrals from medical professionals, web-based media, or word-of-mouth, as previously described^2^. This study was approved by the Research Ethics Board (REB) at The Hospital for Sick Children (REB # 0019980189) and informed consent was obtained from participants or their legal guardians, when appropriate. A total of 253 probands, 322 siblings (including 288 infant siblings), and 447 parents (242 mothers; 205 fathers) constituted the final cohort and were analyzed.

All families had at least 1 child (i.e., the proband) diagnosed with ASD according to the Diagnostic and Statistical Manual for Mental Disorders, 4th Edition (DSM-IV). We excluded probands with a genetic syndrome that could account for their ASD (e.g., Fragile-X, Rett Syndrome)^50^. Younger siblings were recruited at a mean age of 10.2 ± 8.4 months; we followed them, and determined their clinical outcomes with respect to ASD at a mean age of 37.4 ± 2.3 months. An expert clinician determined diagnostic status by clinical best-estimate, informed by the Autism Diagnostic Observation Schedule (ADOS) (calibrated severity score (CSS) ≥ 4 as threshold)^51^, DSM-IV criteria, psychometric assessment of language and cognitive development (Mullen Scales of Early Learning (MSEL)^52^), adaptive functioning (Vineland Adaptive Behavioral Scales (VABS)^36^), and overall clinical impression.

Adapting criteria from published reports of this cohort^6^, from among infant siblings not diagnosed with ASD, we defined another subgroup as “atypically developing”. This classification was based on the presence of elevated ASD symptoms (CSS ≥ 3, requisite scores on the Autism Diagnostic Index-Revised and clinical impression), developmental delay (MSEL composite score > 1 SD below the mean), language delay (MSEL expressive and/or receptive language subscale score > 1 SD below the mean), deficits in adaptive behavior (VABS composite score or subscale score > 1 SD below the mean) or other atypical behavior patterns (e.g., externalizing behaviors) or disorders (e.g., ADHD), as indicated by the clinician best-estimate diagnoses and/or scores above cutoff.

From 253 families, 144 mothers and 127 fathers self-reported the Broader Autism Phenotype Questionnaire (BAPQ) (Supplementary Table 8). The BAPQ includes items mapping onto 3 broader scales (Aloof, Rigid, and Pragmatic Language), which comprise ASD-related subclinical traits previously reported in a subset of parents. A cutoff of 3.15 was shown to optimize agreement with expert clinical assessment of the Broader Autism Phenotype (BAP)^53^.

### Baby Siblings Research Consortium (BSRC) sample collection

For microarray analysis, biological samples were obtained from 251 probands, 321 siblings and 444 parents (241 mothers; 203 fathers) at their respective recruiting sites (total samples = 1016). Blood samples from U.S. sites (*n* = 517) were submitted to the DNA and Cell Repository at Rutgers University, and extracted DNA was subsequently sent to The Center for Applied Genomics (TCAG). Genomic DNA used for genotyping on microarray was extracted from whole blood (86.0%; 874/1016 samples), saliva (0.4%; 4/1016), lymphoblastoid cell lines (10.4%;106/1016), or source undocumented (3.1%; 32/1016).

### Microarray genotyping quality control metrics

Genomic DNA samples were processed on the high-density Affymetrix CytoScan^TM^ HD microarray platform at TCAG (2.67 million copy number markers) following protocols in our other published studies^10,17,18,54^. Quality control thresholds were imposed, as specified by the manufacturer: Waviness standard deviation (Waviness SD) ≤ 0.12; median absolute pairwise difference (MAPD) ≤ 0.25; and single-nucleotide polymorphism (SNP) quality control (SNP QC) ≥ 15.0. One father’s sample did not meet these criteria, but the sample was retained in the analysis to help identify CNV segregation. We used PLINK^55^ software to identify loss of heterozygosity (LOH) and Mendelian inconsistencies using the 750,000 informative SNPs available on the array. We observed Mendelian inconsistencies in 3 families, and eliminated them from the study.

### Variant detection and characterization

We detected CNVs using 4 algorithms: Chromosome Analysis Suite (ChAS) (Affymetrix Inc., USA), iPattern^56^, Nexus^57^, and Partek^58^. We sequentially applied a series of data constraints to ascertain high-confidence, rare CNVs^27^. We considered only stringent CNVs, called by ≥2 algorithms, at least 1 of which was ChAS or iPattern, for downstream analyses. CNVs on the X chromosome were called uniquely by ChAS and iPattern. We eliminated all calls on the Y chromosome. We retained CNVs ≥ 15 kb in length and overlapping ≥10 consecutive probes to reduce the detection of false-positive calls. They were then restricted to those in which ≤70% spanned a segmental duplication and ≥75% of the variant was present in a copy number stable region as previously defined^59^. We used a platform-matched control dataset consisting of 873 individuals with no reported psychiatric history, from the Ontario Populations Genomics Platform (OPGP)^60^, to classify CNVs as rare. We considered a CNV as rare if it did not exceed 50% reciprocal overlap with a CNV found in <0.1% of the control dataset. To corroborate a CNV’s status as rare, we compared to an additional 4 unrelated control populations totaling 9978 individuals: the Collaborative Genetic Study of Nicotine Dependence (COGEND)^61^ and KORA^62^, genotyped on the Illumina Omni 2.5 M; the SAGE consortium controls^63^, Ontario Colorectal Cancer case-control study cohort^64,65^ and the Health, Aging, and Body Composition (Health ABC) Study^66^, genotyped on the Illumina 1 M; the Ottawa Heart Institute controls^67^, and POPGEN^68^, both genotyped on the Affymetrix 6.0 microarray. We sequenced whole genomes of 84 probands, 118 infant siblings and 158 parents (86 mothers; 72 fathers), from 91 families, using DNA from whole blood^17^.

We used criteria adapted from the American College of Medical Genetics classification^69^ and an established annotation strategy^14,18,70^ to classify CNVs as ASD-relevant and pathogenic, likely pathogenic or variant of unknown significance (VUS). A CNV was considered pathogenic or likely pathogenic if (a) it was associated with an established genomic disorder of which ASD is a characteristic (e.g., 16p11.2 microdeletion), or (b) it overlapped a coding exon of a high-confidence ASD-susceptibility gene (e.g., *SHANK3*; Supplementary Data File 3). We considered whether the CNV overlapping the gene was de novo (pathogenic) or inherited (likely pathogenic). Variants overlapping exons of long noncoding RNA *PTCHD1-AS*^71^, and specific noncoding exons of the *MBD5* gene^72^, which constitute the critical region of 2q23.1 microdeletion syndrome, were retained in the analysis. We further defined a class of VUS as ASD-relevant if they overlapped exons of candidate ASD-susceptibility genes or related neuropsychiatric disorder genes (Supplementary Data File 3) and had a frequency ~0.1% in the Database of Genomic Variants. We assessed the efficacy of the variants so identified, as markers for ASD or atypical phenotype status, using the epiR package in R^73^ to calculate positive predictive value, negative predictive value, sensitivity and specificity. All CNVs in infant siblings were classified while blinded to the phenotype status of the individual.

Whole-genome sequence data were processed as previously described^17^. We defined rare loss of function and de novo damaging missense variants as in Yuen et al.^10^. We prioritized single-nucleotide variants and indels that overlapped genes associated with ASD and other related neurodevelopment disorders, and considered whether variants with similar transcriptional consequences were found in other ASD cases.

### Molecular validation and characterization of CNVs

The presence of de novo and ASD-relevant CNVs was confirmed via real-time quantitative PCR (qPCR) using the TaqMan^©^ Copy Number Assay and SYBR^®^ Green methods; all experiments were conducted in triplicate. For SYBR^®^ Green assays, an amplicon 90–140 base pairs in length was amplified using 2 sets of primers positioned ≥ 500 bp from both reported breakpoints. A similar amplicon designed within the *FOXP2* locus served as a 2-copy control^74^. All TaqMan^©^ Copy Number Assays involved predesigned probes located in the gene of interest and *RNaseP*, which served as an endogenous control. All experiments included both male and female control samples (HapMap samples: NA10851 (male) and NA15510 (female)).

### Simons Simplex Collection (SSC) to assess CNV false discovery

In order to assess the possible false discovery for CNVs associated with ASD, we performed a separate CNV analysis on 2110 families from the SSC^33^. This included 2107 mothers, 2107 fathers, 2124 ASD probands, and 2,425 siblings. Families were recruited as previously described (Fischbach and Lord, 2010). Of the 2425 sibs, 2093 are designated unaffected. Of the remaining 332 siblings, 2 have ASD and have thus been excluded from the analysis, leaving 2423 sibs unaffected by ASD available for CNV analysis.

As we did for unaffected sibs in the BSRC cohort of infant siblings, we looked for atypical developmental outcomes in these non-ASD sibs using the psychometric tools available. We considered the potential presence of ASD traits using the Social Responsiveness Scale (SRS)-Parent Report (*n* = 2298)^34^; mild-moderate developmental delay by identifying adaptive behavior deficits using the VABS (*n* = 2368) and other emotional and behavioral concerns as identified through the Child Behavioral Checklist (CBCL)-Parent Report^35^ (*n* = 448 for ages 1.5–5 test; *n* = 1833 for ages 6–18 test). To define atypical development, we applied a cutoff of >1 SD below the mean for the total scores for the SRS and the CBCL, expressed as *T*-scores (i.e., *T*-scores > 60), and the Adaptive Behavior Composite from the VABS, expressed as a standard score (i.e., scores < 85). Sibs had to meet this cutoff on at least 1 of the 3 tests to qualify as atypically developing.

We received microarray intensity data in the form of .IDAT files for 8761 individuals genotyped on 3 different microarray platforms: 1246 on the Illumina Human1Mv1; 3826 on the Illumina Human1M-Duov3; and 3689 on the Illumina HumanOmni2.5–4v1. We called CNVs using the same pipeline as for the infant sibling cohort, with the exception that the following 3 algorithms were employed, as previously described:^18^ PennCNV^75^, QuantiSNP^76^, and iPattern. Stringent CNVs were called by a minimum of 2 of these algorithms, with at least one being iPattern. We applied criteria for filtering and prioritizing variants as described above. CNVs were not molecularly characterized, as no DNA for this cohort was available.

### Polygenic transmission disequilibrium test

We analyzed the contribution of common genetic variants to ASD risk for families from the infant sibling cohort for which microarray data were available for the proband (*n* = 189), at least 1 infant sib (*n* = 193) and both parents. We generated PRS using PRSice^77^ (clump-kb 250, clump-p 1.000000, clump-r2 0.100000, info-base 0.9), using a *p*-value threshold of 0.1 as suggested by Grove et al.^78^ in Supplemental Fig. 4.4.1. In total, 246,607 SNPs were included in both the SNP genotypes from the Affymetrix CytoScan^TM^ HD array and the iPSYCH-PGC_ASD_Nov2017 GWAS summary statistic file, with 9,112,386 variants, of which 17,362 were removed due to having an INFO score less than 0.90. For the study genotypes, we began with 749,157 SNPs from the Affymetrix CytoScan^TM^ HD array, removing 83,849 SNPs with a call rate >90% and 97,770 SNPs with minor allele frequency < 5%. Using PRSice, 87,215 genotype array variants were removed (e.g., A- > T), with 228,151 intersecting the ASD GWAS variants. A total of 52,923 SNPs remained after linkage disequlibrium-based clumping, of which 11,401 met the specified *p*-value threshold, and were thus used for PRS computation.

We performed the same analysis for 2104 families from the Simons Simplex Collection. All families consisted of 2x parents (*n* = 2104 mothers; *n* = 2104 fathers), a proband (*n* = 2118) and at least 1 unaffected sibling (*n* = 2416). Using identical criteria for inclusion of SNPs, 53,803 SNPs remained after linkage disequilibrium-based clumping, and 11,161 SNPs were enriched for in cases versus controls as per a *p*-value of 0.1, and therefore used for the generation of PRS.

Previous estimates for narrow-sense heritability (h2g) for ASD are varied, depending on phenotype definition, study design, genotyping method, and other factors. Estimates include 12%^37^, 52%^79^, and 83%^80^; we used h2g = 60% in the power calculation. Power was calculated under the liability threshold model assuming prevalence of ASD of 1.5%^81^.

### Reporting summary

Further information on research design is available in the Nature Research Reporting Summary linked to this article.

## Supplementary information

Supplementary Information

Supplementary Dataset 1

Supplementary Dataset 2

Supplementary Dataset 3

Reporting Summary

Description of Additional Supplementary Files

## Data Availability

All raw microarray data for 1016 individuals that were genotyped on the Affymetrix CytoScan^TM^ HD Array were submitted to dbGaP and can be accessed via a data access committee using dbGaP accession number phs001876.v1.p1. Whole-genome sequence data are accessible through the Autism Speaks MSSNG database (https://research.mss.ng).

## Source data

Source Data
