# Supplementary material for: Predictive impact of rare genomic copy number variations in siblings of individuals with autism spectrum disorders
Source: Nat Commun. 2019 Dec 5;10:5519. doi: 10.1038/s41467-019-13380-2 (PMC6892938; doi:10.1038/s41467-019-13380-2)
Supplement: Supplementary file 1 — Supplementary Information [file 41467_2019_13380_MOESM1_ESM.pdf]

Supplementary Information

"Predictive impact of rare genomic copy number variations in siblings of individuals with autism spectrum disorders"

D'Abate et al.

Supplementary Information

Table of Contents

Supplementary Figure 1...………………………………………………………….... 3

Supplementary Figure 2...………………………………………………………….... 4

Supplementary Figure 3...………………………………………………………….... 5

Supplementary Table 1..………………………………………………........................ 6

Supplementary Table 2..………………………………………………........................ 7

Supplementary Table 3..………………………………………………........................ 8

Supplementary Table 4..………………………………………………........................ 9

Supplementary Table 5..………………………………………………....................... 10

Supplementary Table 6..………………………………………………....................... 11

Supplementary Table 7..………………………………………………....................... 12

Supplementary Table 8..………………………………………………....................... 13

Supplementary Note 1………………………………………………………………... 14

Supplementary References……………………………………………………………. 17

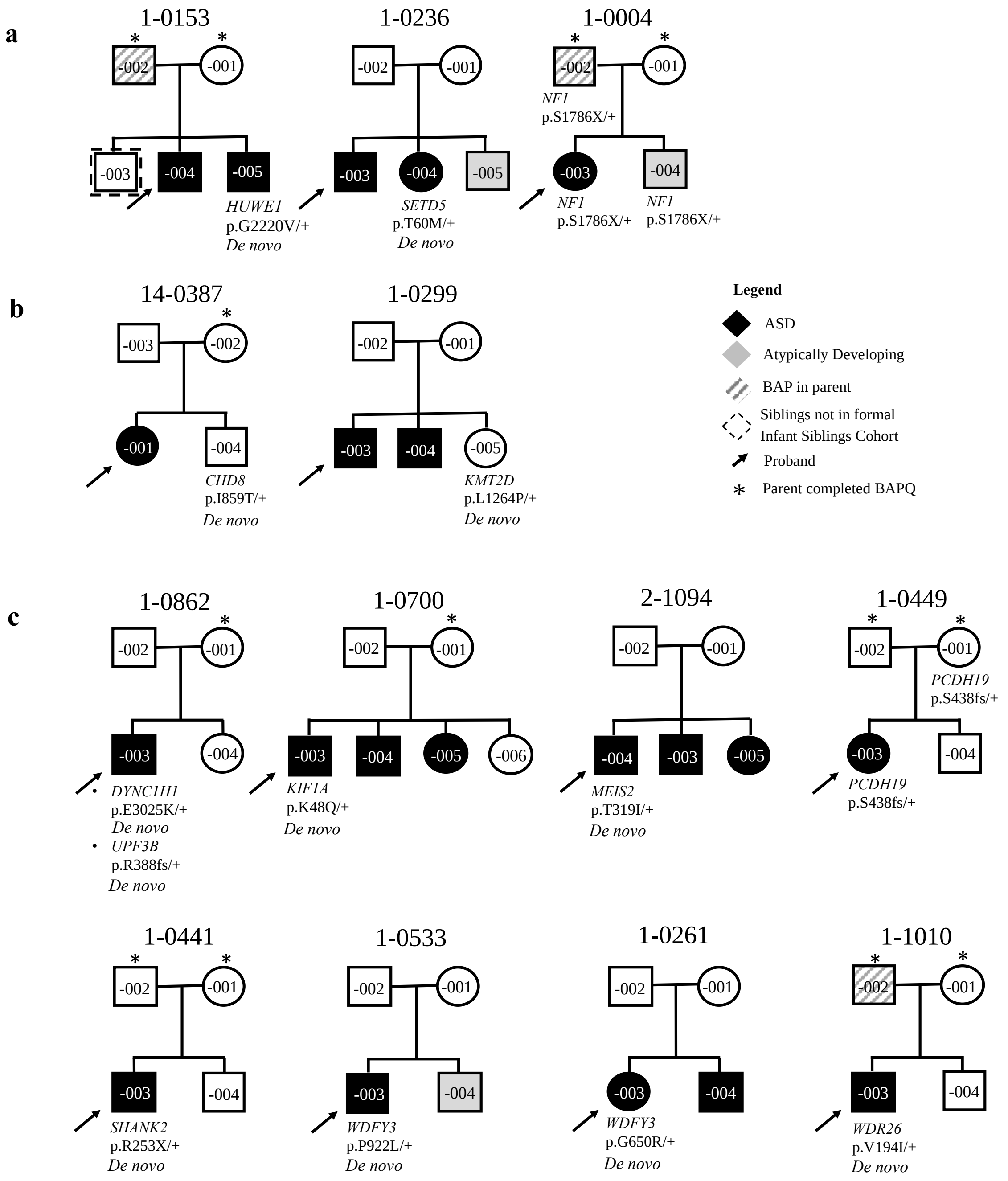

Supplementary Figure 1. Single-nucleotide variants and indels in probands and infant siblings

a. ASD-relevant SNVs in infant siblings with ASD or who were atypically developing. One variant (family 1-0004) is shared between a proband and atypically developing sibling.

b. ASD-relevant SNVs in typically developing infant siblings.

c. ASD-relevant SNVs and indels in probands.

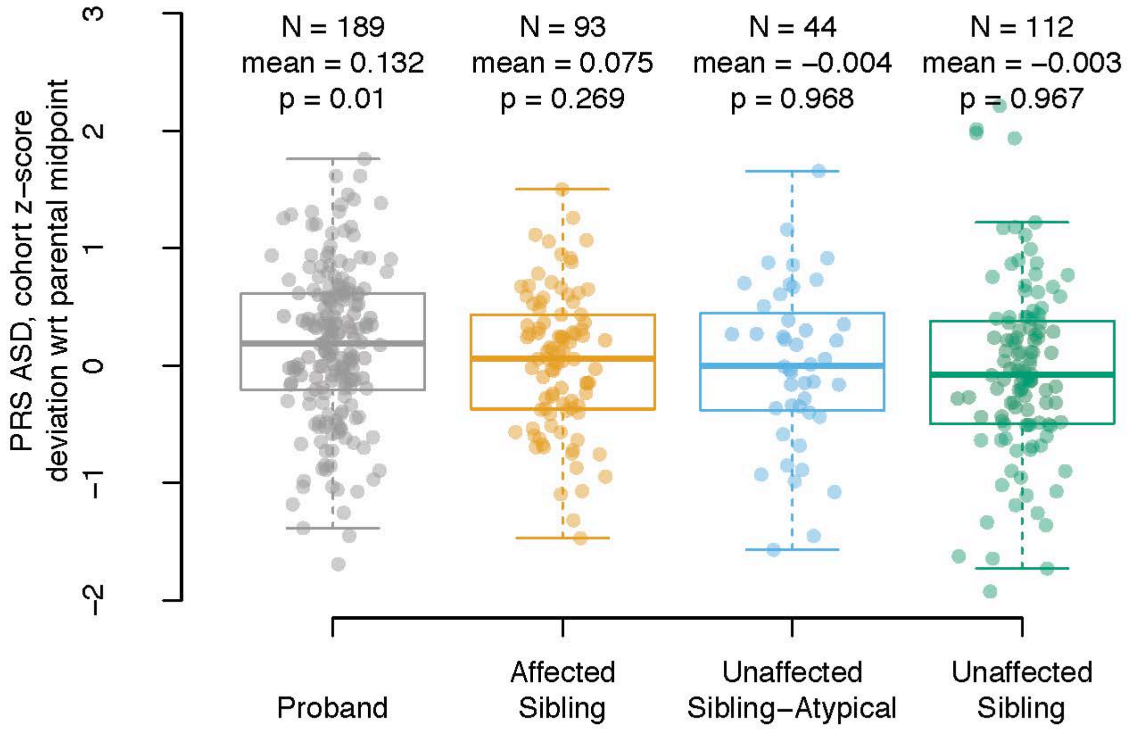

Supplementary Figure 2. Polygenic risk scores in probands and infant siblings

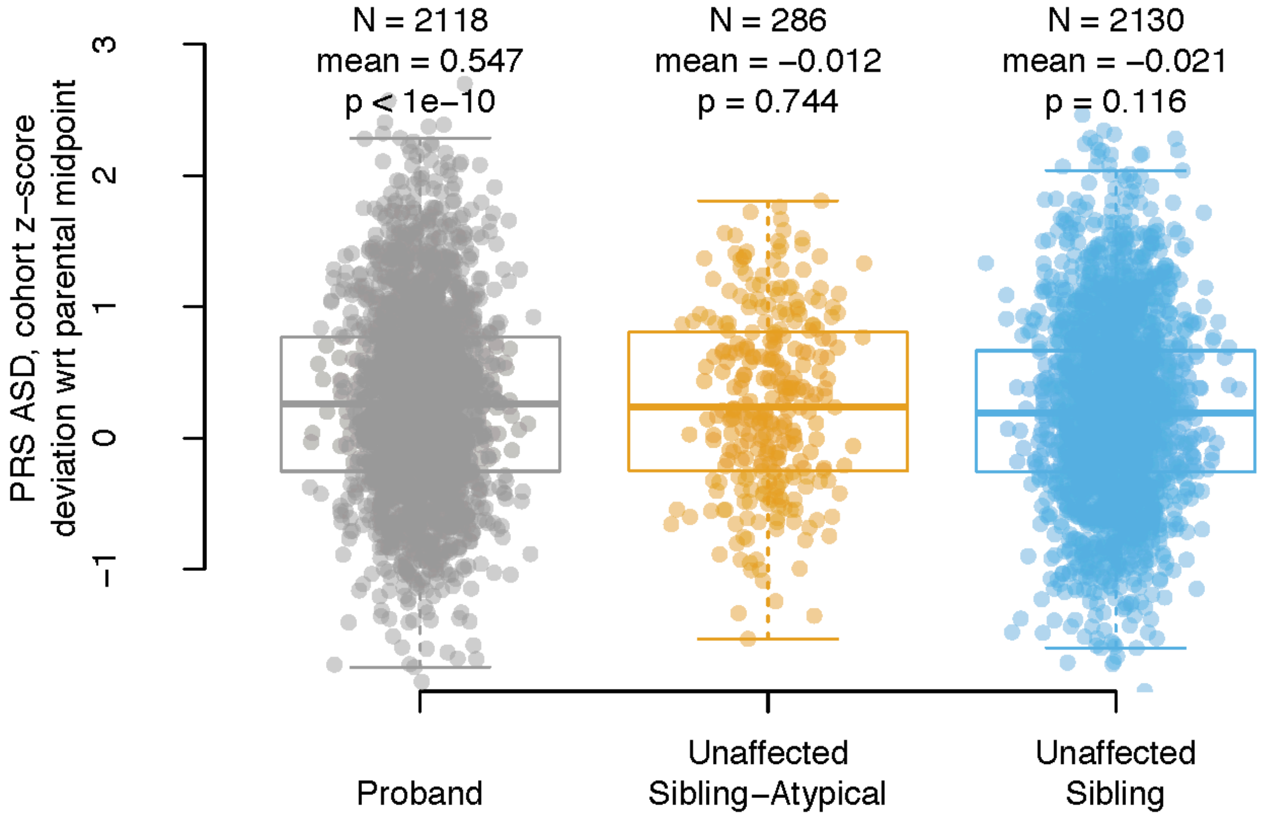

Supplementary Figure 3. Polygenic risk scores in probands and siblings from the Simons Simplex Collection

Supplementary Table 1: Cohort descriptive statistics

	%	Counts	Age at ascertainment (±SD)
Probands, male	82.6	209 (of 253)	
Affected infant siblings, male	78.6	81 (of 103)	
Atypically developing infant siblings, male	61.1	33 (of 54)	
Typically developing infant siblings, male	53.4	72 (of 131)	
Multiplex families (at ascertainment)	37.1	94 (of 253)	
Mean age of infant sibling at first visit (months)		206 (of 288*)	10.2 (±8.4)
Mean maternal age at infant sibling's birth (years)		141 (of 242†)	34.6 (±5.3)
Mean paternal age at infant sibling's birth (years)		140 (of 205†)	37 (±6.2)

* Age of infant siblings at first visit only available for 206 individuals.
† Parental age at sibling's birth only available 141 mothers and 140 fathers, respectively.

Supplementary Table 2: Psychometric assessment summary table

	Affected siblings (n=103)				Atypically developing siblings (n=54)				Typically developing siblings (n=131)			
		Mean	SD	Range		Mean	SD	Range		Mean	SD	Range
**Autism Diagnostic Observation Schedule (ADOS)**												
Social Affect Severity Score	n = 51	6.31	1.79	1-10	n = 42	2.14	1.12	1-5	n = 53	2.08	1.21	1-6
Restricted, Repetitive Behaviours Severity Score	n = 66	7.74	2.11	1-10	n = 54	4.76	2.52	1-9	n = 77	3.48	2.67	1-9
Calibrated Severity Score	n = 49	7.04	2.01	1-10	n = 40	2.13	1.24	1-5	n = 53	1.75	1.09	1-6
**Autism Diagnostic Interview - Revised (ADI-R)**												
Reciprocal Social Interaction	n = 32	12.13	5.80	1-22	n = 21	4.38	3.22	0-11	n = 25	3.32	3.66	0-16
Communication (verbal)	n = 24	9.88	4.25	2-18	n = 20	4.30	3.25	0-13	n = 25	2.56	2.72	0-13
Communication (non-verbal)	n = 9	11.11	1.69	7-13	n = 1	3	N/A	N/A	n = 1	9	N/A	N/A
Restricted, Repetitive Behaviours and Stereotypy	n = 32	4.50	2.86	0-10	n = 21	1.57	1.86	0-6	n = 25	0.96	1.27	0-4
Developmental Abnormality	n = 33	2.79	1.52	0-5	n = 21	1.38	1.12	0-4	n = 25	0.68	0.99	0-3
**Child's Behavioural Checklist (CBCL)**												
Internalizing T-Score		52.77	12.48	29-84		44.37	11.05	29-70		39.82	10.62	29-70
Externalizing T-Score		56.23	11.58	32-86		48.47	12.43	28-82		42.96	8.35	32-59
Affective T-Score		55.83	8.10	50-84		53.21	5.46	50-67		51.21	2.42	50-63
Anxiety T-Score	n = 35	53.74	6.32	50-81	n= 19	54.05	7.71	50-79	n= 28	51.68	3.04	50-60
ADHD T-Score		56.91	7.41	50-76		53.21	5.01	50-71		51.32	2.09	50-57
Odd T-Score		56.54	7.56	50-80		55.11	8.72	50-80		51.82	3.67	50-67
Total Problems T-Score		55.69	11.90	31-90		47.05	10.68	28-65		41.46	8.77	30-65
**Mullen Scales of Early Learning (MSEL)**												
Early Learning Composite	n = 56	86.16	23.90	49-137	n = 50	89.46	17.55	56-127	n = 88	109.32	15.89	67-150
Visual Reception T-Score	n = 62	44.08	18.07	20-80	n = 51	50.25	15.12	20-80	n = 95	61.43	11.28	36-80
Fine Motor T-Score	n = 64	37.28	13.87	20-71	n = 51	43.00	12.22	20-67	n = 95	51.27	12.75	20-80
Receptive Language T-Score	n = 57	40.58	13.73	19-75	n = 52	42.33	10.26	20-70	n = 95	50.96	8.48	25-71
Expressive Language T-Score	n = 60	41.47	13.71	19-65	n = 52	43.98	10.81	23-70	n = 94	54.02	8.73	21-80
**Vineland Adaptive Behavioural Scales (VABS)**												
Adaptive Behaviour Composite	n = 40	81.40	13.75	55-116	n = 37	94.14	13.72	69-133	n = 57	99.88	8.06	82-118
Communication Standard Score	n = 44	88.25	16.47	57-120	n = 40	96.40	12.29	68-123	n = 60	105.43	9.69	87-132
Daily Living Standard Score	n = 40	79.68	13.20	53-111	n = 37	92.08	16.39	23-121	n = 57	97.19	11.20	58-121
Socialization Standard Score	n = 46	86.33	15.07	60-118	n = 40	95.58	11.85	73-120	n = 66	99.86	9.30	81-120
Motor Standard Score	n = 46	81.40	13.75	55-116	n = 37	96.76	13.78	75-146	n = 57	97.28	11.03	77-143

Supplementary Table 3: *De novo* CNVs in all subjects

Individual ID	Sex	Status	Family type	Cytoband	#:start-end (hg19)	Size	CNV type	Genes
1-0083-003	M	proband	simplex	7q31.1-7q.31	7:108606430-119657890	11.1 Mb	loss	[1]*FOXP2;EIF3IP1;MIR6132;IMMP2L;LOC100996249;LINC01393;CTTNBP2;DOCK4;LINC00998;LINC01510; LSMEM1;CAV2;CAV1;ANKRD7;ST7-AS1; PPP1R3A;LINC01392;ST7;TES;GPR85;ST7-OT3;LSM8;TFEC; ST7-OT4; MDFIC; ASZ1; DOCK4-AS1;IFRD1;MIR3666;TMEM168;CAPZA2;ST7-AS2; C7orf60; WNT2;LRRN3;MET; ZNF277;LOC101928036; CFTR;LOC101928012*
14-0139-001	F	proband	simplex	19q13.33	19:48462617-51107899	2.6 Mb	gain	*MIR4751;LIN7B;SNAR-D;SIGLEC11;MAMSTR;CD37;IZUMO1;MED25;PIH1D1;MYH14;ASPDH;NOSIP; NAPSA;NAPSB;RPL18;GRWD1;SNAR-G2;LIG1;ADM5; ZNF473; ELSPBP1;LHB; VRK3;PNKP;MIR4749; PRMT1; BCAT2;EMC10;SIGLEC16;ALDH16A1;LRRC4B;MIR6800;CCDC155; MIR6799;NUCB1; HRC; MIR4750;LMTK3; MIR6798; PRRG2;MIR150;IZUMO2;PLEKHA4;DKKL1;DBP;BSPH1;HSD17B14;PTOV1;FUZ; SNORD35A; SNORD35B;SYNGR4; RRAS; PRR12;RUVBL2;TULP2;SEC1P;PTOV1-AS2; C19orf68;PTOV1-AS1;SNAR-G1; CARD8;AKT1S1;TRPM4;NR1H2;RCN3;SNORD32A;SNAR-A14;SNAR-A11; SNAR-A10;GYS1;PTH2;FTL; SPACA4;RPL13AP5;KDELR1; NUCB1-AS1;CABP5;TBC1D17;MYBPC2;SULT2B1; ZNF114;FGF21;RASIP1;DHDH; KCNA7; C19orf73;NUP62;POLD1;CGB;RPL13A;SNAR-A9;SNAR-A8;SNAR-A3; IL4I1; SNRNP70;SNAR-A7; SLC6A16; SNAR-A5;SNAR-A4;IRF3;NTF4;TSKS; CGB5; CGB7;CGB1;CGB2;SPIB;SNAR-B1; FLT3LG;SCAF1; JOSD2; CCDC114; SLC17A7; NTN5; SNAR-A6;PPP1R15A;EMP3;CARD8-AS1; SPHK2; RPS11; MIR4324; KCNC3;BAX;TEAD2;CGB8; FAM83E;FLJ26850; PPFIA3;BCL2L12; LOC101059948;FAM71E1;MIR5088; FCGRT;CPT1C;FUT1; KCNJ14;CA11; LOC101928295; AP2A1;PLA2G4C;CYTH2;SNAR-B2;GFY;SNORD34; SNORD33;FUT2;GRIN2D; ATF5;TMEM143*
12-4453-005	M	atypically developing sibling	simplex	15q21.3-15q22.2	15:57859275-59410122	1.6 Mb	gain	*CCNB2;LIPC;FAM63B;SLTM;GCOM1;HSP90AB4P;AQP9;ADAM10;LOC101928694;MYZAP;RNF111;POLR2M; ALDH1A2*
14-0387-001	F	proband	simplex	5p14.3	5: 18767377-20113902	1.4 Mb	Loss	*CDH18*
1-0298-003	M	proband	simplex	17q25.3	17:79330617-80189678	859 kb	gain	[2]*C17orf70;ACTG1;TSPAN10;ANAPC11;OXLD1;STRA13;RFNG;ARL16;MIR3186;NPLOC4;PYCR1;SLC25A10;GPS1; DUS1L;MIR6786;MAFG-AS1;DCXR;FASN; LOC100130370;ARHGDIA;MAFG;BAHCC1;MIR4740;MRPL12;SIRT7; RAC3;CCDC57;P4HB;PCYT2;HGS;ALYREF;GCGR;MYADML2;FSCN2; ASPSCR1;CCDC137; NOTUM;FAM195B; SLC16A3;NPB;PPP1R27;PDE6G;LRRC45*
					17:80190109-80252756	63 kb	loss	[2]*LINC01970;SLC16A3;CSNK1D;MIR6787*
14-0376-004	M	typically developing sibling	simplex	16p11.2	16:29567296-30177928	610 kb	loss	*DOC2A;ASPHD1;TBX6;PRRT2;CDIPT;QPRT;SMG1P2;YPEL3;SLC7A5P1;PPP4C;MAPK3;SPN;MVP;FAM57B;ZG16; ALDOA;INO80E;SEZ6L2;TAOK2;KCTD13; MAZ; KIF22;GDPD3;C16orf92;C16orf54;CDIPT-AS1; TMEM219;PAGR1;HIRIP3*
4-0040-003	F	proband	multiplex	18p11.32	18:380185-926925	547 kb	gain	*C18orf56,CETN1, TYMS, CLULI, COLEC12, YES1, ADCYAP1, ENOSF1*
15-1126-001	F	proband	multiplex	7q36.3	7:155124600-155465851	341 kb	gain	*RBM33;BLACE;EN2; CNPY1;LOC100506302*
14-0258-001	F	proband	simplex	2q32.1-2	2:189149450-189477881	328 kb	loss	*GULP1, LINC01090, MIR561*
14-0243-004	F	typically developing sibling	simplex	15q22.31	15:64721928-65000963	279 kb	loss	*TRIP4, ZNF609, OAZ2*
14-0384-001	F	proband	simplex	16p11.2	16:28808206-29051191	243 kb	loss	*ATXN2L;ATP2A1;NFATC2IP;ATP2A1-AS1;MIR4721;SPNS1;RABEP2;SH2B1;LAT;MIR4517;TUFM;CD19*
1-0616-005 (non-infant sib)	M	typically developing sibling	simplex	5p15.33	5:1282492-1474755	192 kb	gain	*SLC6A3;LINC01511;CLPTM1L;TERT;MIR4457;LPCAT1*
1-0514-003	M	proband	simplex	7p22.1	7:5392787-5522717	130 kb	loss	*FBXL18, TNRC18*
1-0514-004	F	atypically developing sibling	simplex	Xq13.1	X:68321236-68450191	129 kb	gain	*PJA1, LINC00269*
14-0119-001	M	proband	simplex	1q21.3	1:151144910-151243214	98 kb	loss	*PSMD4, TMOD4, PIP5K1A, VPS72*
14-0356-004	F	atypically developing sibling	simplex	1q22	1:155561901-155636887	75 kb	loss	*MSTO1, MSTO2P, Y1AP1*
14-0033-004	M	typically developing sibling	simplex	2q22.1	2:141414593-141432376	17.8 kb	loss	*LRP1B*

1. Published by Feuk *et al.* (2006)
2. Published by Pinto *et al.* (2010)

Supplementary Table 4: Clinically-relevant CNVs in probands (n=253), ASD affected siblings (n=103), atypically developing siblings (n=54) and unaffected siblings (n=131)

Individual ID	Sex	Status	Family Type	Inheritance	Cytoband	#:Start-End (hg19)	Size	Variant type	Diagnostic Classification	Genes
1-0083-003	M	proband	simplex	*de novo*	7q31.1- 7q.31	7:108606430-119657890	11.1 Mb	loss	Pathogenic	[1]*FOXP2;EIF3IP1;MIR6132;IMMP2L;LOC100996249;LINC01393;CTTNBP2; DOCK4;LINC00998;LINC01510; LSMEM1;CAV2;CAV1;ANKRD7;ST7-AS1; PPP1R3A;LINC01392;ST7;TES;GPR85;ST7-OT3; LSM8;TFEC;ST7-OT4; MDFIC;ASZ1;DOCK4-AS1;I FRD1;MIR3666;TMEM168;CAPZA2; ST7-AS2; C7orf60; WNT2;LRRN3;MET;ZNF277; LOC101928036;CFTR; LOC101928012*
10-1076-004	M	ASD sibling	multiplex	maternal	22q11.21	22: 18644791-21465659<br>(22q11.2 duplication syndrome)	2.8 Mb	gain	Pathogenic	*FAM230A;RIMBP3;TMEM191A;LINC00896;PI4KA;LINC00895;AIFM3;SLC7A4;SNORA77B;MIR185;GNB1L;TBX1;MIR3618;TANGO2;MIR1306;SEPT5;LOC102725 072;GP1BB;THAP7;ZNF74;P2RX6;DGCR9;PRODH;BCRP2;CRKL;C22orf29;TMEM191B;MIR649;DGCR2;DGCR5;LINC01637;DGCR6;C22orf39;LINC01311;DGC R6L;MED15;UFD1;TXNRD2;CLDN5;RTN4R;TSSK2;GSC2;ARVCF;SLC25A1;MIR4761;COMT;LOC284865;P2RX6P;CDC45; THAP7AS1;DGCR8; KLHL22; CLTCL1;USP18;LINC01660;SERPIND1;LRRC74B;LOC105379550;MIR6816;LOC101927859;SCARF2;HIRA;CCDC188; MIR1286;RANBP1;GGT3P;POM121L4P; SNAP29; PI4KAP1;DGCR11;DGCR10; MRPL40;TUBA3FP;DGCR14;ZDHHC8;LINC01662;LINC01663;TRMT2A;LZTR1; LOC100996415;SEPT5-GP1BB*
14-0139-001	F	proband	simplex	*de novo*	19q13.33	19:48462617-51107899	2.6 Mb	gain	VUS	*MIR4751;LIN7B;SNAR-D;SIGLEC11;MAMSTR;CD37;IZUMO1; MED25; PIH1D1; MYH14; ASPDH;NOSIP; NAPSA;NAPSB;RPL18; GRWD1;SNARG2; LIG1;ADM5; ZNF473;ELSPBP1;LHB;VRK3; PNKP; MIR4749; PRMT1;BCAT2;EMC10; SIGLEC16;ALDH16A1; LRRC4B;MIR6800; CCDC155; MIR6799;NUCB1;HRC; MIR4750; LMTK3;MIR6798;PRRG2;MIR150;IZUMO2;PLEKHA4;DKKL1;DBP; BSPH1;HSD17B14;PTOV1; FUZ; SNORD35A;SNORD35B;SYNGR4; RRAS; PRR12; RUVBL2; TULP2;SEC1P;PTOV1-AS2;C19orf68;PTOV1-AS1; SNAR-G1; CARD8;AKT1S1; TRPM4;NR1H2;RCN3; SNORD32A;SNAR-A14;SNAR-A11;SNAR-A10; GYS1;PTH2; FTL;SPACA4;RPL13AP5;KDELR1; NUCB1AS1;CABP5;TBC1D17; MYBPC2; SULT2B1; ZNF114; FGF21;RASIP1; DHDH;KCNA7;C19orf73; UP62; POLD1;CGB; RPL13A;SNAR-A9;SNAR-A8;SNAR-A3;IL4I1;SNRNP70;SNAR-A7; SLC6A16;SNAR-A5; SNAR-A4;IRF3;NTF4;TSKS; CGB5;CGB7;CGB1;CGB2;SPIB;SNAR-B1; FLT3LG; SCAF1; JOSD2;CCDC114;SLC17A7;NTN5; SNARA6;PPP1R15A; EMP3;CARD8-AS1; SPHK2;RPS11; MIR4324; KCNC3;BAX;TEAD2;CGB8; FAM83E; FLJ26850;PPFIA3; BCL2L12; LOC101059948;FAM71E1; MIR5088; FCGRT; CPT1C;FUT1; KCNJ14; CA11; LOC101928295; AP2A1; PLA2G4C;CYTH2;SNAR-B2; GFY;SNORD34; SNORD33;FUT2;GRIN2D;ATF5;TMEM143*
4-0061-004	M	atypically developing sibling	simplex	paternal	3p26.3-2	3:1080020-2886098	1.8 Mb	loss	VUS	*CNTN6,CNTN4,CNTN4-AS2*
12-8115-001<br>12-8115-004	M<br>M	proband<br>typically developing sibling	simplex	maternal	16p13.11	16: 14866284-16529801<br>(16p13.11 microduplication syndrome)	1.7 Mb	gain	VUS	*LOC100288162;MYH11;MIR484;MARF1;NTAN1;FOPNL;NDE1;ABCC1;PKD1P4-NPIPA8; MIR6511A3;MIR6506; PKD1P1;PKD1P3-NPIPA1;ABCC6P2;MIR3670-4; MIR6511A2;MIR6511A1;MIR3670-1;MIR3670-2; MIR6511A4; C16orf45;LOC100505915;MIR3670-3;PKD1P6-NPIPP1;NOMO3;NOMO1;MIR3179-2; MIR3179-3; MIR3180-4; MIR3179-1;MIR3180-2;MIR3180-3;MIR3179-4;MIR3180-1;NPIPA7;PDXDC1;RRN3; MIR6511B2; MPV17L; NPIPA1;NPIPA8;NPIPA5;ABCC6; MIR6511B1;MIR6770-2;MIR6770-3;MIR6770-1*
12-4453-005	M	atypically developing sibling	simplex	*de novo*	15q21.3-15q22.2	15:57859275-59410122	1.6 Mb	gain	VUS	*CCNB2;LIPC;FAM63B;SLTM;GCOM1;HSP90AB4P;AQP9;ADAM10; LOC101928694;MYZAP;RNF111;POLR2M;ALDH1A2*
4-0027-003<br>4-0027-004	M<br>F	proband<br>atypically developing sibling	simplex	maternal	15q13.1-2	15:29010256-30386398	1.4Mb	gain	VUS	*LOC100289656;TJP1;APBA2;GOLGA8J;GOLGA6L7P;PDCD6IPP2;NDNL2; FAM189A1*
1-0298-003	M	proband	simplex	*de novo*	17q25.3	17:79330617-80189678	859 kb	gain	VUS	[2]*C17orf70;ACTG1;TSPAN10;ANAPC11;OXLD1;STRA13;RFNG;ARL16;MIR3186; NPLOC4;PYCR1; SLC25A10; GPS1;DUS1L;MIR6786;MAFG-AS1; DCXR;FASN;LOC100130370; ARHGDIA;MAFG;BAHCC1;MIR4740;MRPL12; SIRT7;RAC3;CCDC57;P4HB;PCYT2; HGS;ALYREF;GCGR; MYADML2; FSCN2;ASPSCR1; CCDC137;NOTUM; FAM195B; SLC16A3;NPB;PPP1R27;PDE6G;LRRC45*
						17:80190109-80252756	63 kb	loss	VUS	[2]*LINC01970;SLC16A3;CSNK1D;MIR6787*
14-0152-001	M	proband	simplex	N/A	2q23.3	2:152430332-153267017	837 kb	loss	VUS	*FMNL2;ARL5A;STAM2;NEB;CACNB4*
14-0376-004	M	typically developing sibling	simplex	*de novo*	16p11.2	16:29567296-30177928<br>(16p11.2 microdeletion syndrome BP4-5)	610 kb	loss	Pathogenic	*DOC2A;ASPHD1;TBX6;PRRT2;CDIPT;QPRT;SMG1P2;YPEL3;SLC7A5P1;PPP4C; MAPK3;SPN;MVP;FAM57B; ZG16;ALDOA;INO80E;SEZ6L2;TAOK2; KCTD13; MAZ; KIF22;GDPD3;C16orf92;C16orf54;CDIPT-AS1; TMEM219;PAGR1;HIRIP3*
14-0384-001	F	proband	simplex	*de novo*	16p11.2	16:28808206-29051191<br>(16p11.2 microdeletion syndrome BP2-3)	243 kb	loss	Pathogenic	*ATXN2L;ATP2A1;NFATC2IP;ATP2A1-AS1; MIR4721;SPNS1;RABEP2;SH2B1;LAT;MIR4517;TUFM;CD19*
4-0062-003<br>4-0062-004	M<br>F	proband<br>atypically developing sibling	simplex	paternal	2p16.3	2:51141571-51370150	229 kb	loss	Likely Pathogenic	*NRXN1;LOC730100*
1-0616-003<br>1-0616-004<br>1-0616-005 (non-infant sib)	M<br>M<br>M	proband<br>atypically developing sibling<br>typically developing sibling	simplex	maternal	16p11.2	16:28824491-29051191<br>(16p11.2 microdeletion syndrome BP2-3)	227 kb	loss	Pathogenic	*ATXN2L;ATP2A1;NFATC2IP;ATP2A1-AS1; MIR4721;SPNS1;RABEP2;SH2B1;LAT;MIR4517;TUFM;CD19*
12-4168-004<br>12-4168-006 (non-infant sib)<br>12-4168-007 (non-infant sib)	M<br>F<br>M	ASD sibling<br>ASD sibling<br>ASD sibling	multiplex	paternal	2q24.1-2q23.3	2:154881876-155011019	129 kb	loss	VUS	*GALNT13*
12-8257-001<br>12-8257-004<br>12-8257-005 (non-infant sib)	M<br>M<br>M	proband<br>ASD sibling<br>ASD sibling	multiplex	maternal	Xp22.11	X:23043876-23134236	90 kb	loss	Likely Pathogenic	*PTCHD1-AS*
4-0040-003	F	proband	multiplex	*de novo*	18p11.32	18:380185-926925	547 kb	gain	VUS	*C18orf56,CETN1, TYMS, CLULI, COLEC12, YES1, ADCYAP1, ENOSF1*
4-0040-003<br>4-0040-004	F<br>M	proband<br>ASD sibling		maternal	3p26.3	3:2609217-2679515	70 kb	gain	VUS	*CNTN4*
1-0455-003<br>1-0455-004	M<br>M	proband<br>ASD sibling	multiplex	maternal	8p12	8:31991227-32056041	65 kb	loss	VUS	*NRG1;NRG1-IT1*
13-0049-001<br>13-0049-004	M<br>M	proband<br>ASD sibling	multiplex	maternal	2q23.1	2:14093863-149151358	58 kb	loss	Likely Pathogenic	*MBD5*

VUS = variants of unknown significance

1. Published by Feuk *et al.* (2006)
2. Published by Pinto *et al.* (2010)

Supplementary Table 5: Clinically-relevant SNVs and indels in probands (n=84), ASD affected siblings (n= 48), atypically developing siblings (n=14) and typically-developing siblings (n=56)

Individual ID	Sex	Status	Family Type	Inheritance	Genes	#:Start-End (hg19)	Variant type
1-0153-005	M	ASD sibling	multiplex	*de novo*	*HUWE1*	X:53595700	[1]NM_031407: c.G6659T: p.Gly2220Val, het
1-0236-004	F	ASD sibling	multiplex	*de novo*	*SETD5*	3:9476019	NM_001080517: c.C179T:p.Thr60Met, het
1-0004-003	F	proband	simplex	paternal	*NF1*	17:29654605	NM_001042492: c.C5357A: p.Ser1786X, het
1-0004-004	M	atypically developing sibling	simplex	paternal	*NF1*	17:29654605	NM_001042492: c.C5357A: p.Ser1786X, het
14-0387-004	M	typically developing sibling	simplex	*de novo*	*CHD8*	14:21871717	NM_020920: c.T2576C: p.Ile859Thr, het
1-0299-005	F	typically developing sibling	multiplex	*de novo*	*KMT2D*	12:49443580	NM_003482: c.T3791C: p.Leu1264Pro, het
1-0862-003	M	proband	simplex	*de novo*	*DYNC1H1*	14:102493980	[1]NM_001376: c.G9073A: p.Glu3025Lys, het
1-0862-003	M	proband	simplex	*de novo*	*UPF3B*	X:118971855-118971858, del	[1]NM_080632: c.1164_1167del: p.Arg388fs, het
1-0700-003	M	proband	multiplex	*de novo*	*KIF1A*	2:241728694	NM_001244008: c.A142C: p.Lys48Gln, het
2-1094-004	M	proband	multiplex	*de novo*	*MEIS2*	15:37242546	[1]NM_001220482: c.C956T: p.Thr319Ile, het
1-0449-003	F	proband	simplex	maternal	*PCDH19*	X:99662281-99662282, del	NM_001184880: c.1314_1315del: p.Ser438fs, het
1-0441-003	M	proband	simplex	*de novo*	*SHANK2*	11:70336411	[1]NM_133266: c.C757T: p.Arg253X, het
1-0533-003	M	proband	simplex	*de novo*	*WDFY3*	4:85722860	[1]NM_014991: c.C2765T: p.Pro922Leu, het
1-0261-003	F	proband	multiplex	*de novo*	*WDFY3*	4:85731437	[1]NM_014991: c.G1948A: p.Gly650Arg, het
1-1010-003	M	proband	simplex	*de novo*	*WDR26*	1:224619226	NM_025160: c.G580A: p.Val194Ile, het

1. Published by Yuen *et al.* (2017)

Supplementary Table 6: Occurrence of ASD-relevant CNVs (from infant sibling cohort) in probands and unaffected sibs from the Simons Simplex Collection

	# CNVs found in probands (n=2,124)	# CNVs found in non-ASD sibs (n=2,423)
**ASD-Relevant Genes**		
CNTN4/CNTN6	4	8*
MBD5	2	0
NRXN1	5	2
PTCHD1-AS	3	2
**Genomic Disorders**		
16p11.2 microdeletion, distal (SH2B1)	1	0
16p11.2 microdeletion (593 kb)	7	0
16p13.11 microduplication	5	5†
22q11.21 microduplication	3	0

*Atypical female sib with CNTN6 deletion
†Atypical male sib with 16p13.11 duplication

Supplementary Table 7: Predictive statistics of microarray findings for probands and their non-ASD siblings (with or without atypical development) from the Simons Simplex Collection

	ASD (all ASD-relevant CNVs)	ASD + Atypical development (all ASD-relevant CNVs)	ASD (excluding VUS)	ASD + Atypical development (excluding VUS)
Statistic				
Sensitivity (0.95 CI)	0.05 (0.05-0.07)	0.05 (0.04-0.06)	0.04 (0.03-0.05)	0.04 (0.03-0.04)
Specificity (0.95 CI)	0.97 (0.97-0.98)	0.97 (0.97-0.98)	0.99 (0.99-0.99)	0.99 (0.99-0.99)
Positive Predictive Value (0.95 CI)	0.65 (0.57-0.72)	0.69 (0.61-0.75)	0.79 (0.70-0.87)	0.83 (0.74-0.90)
Negative Predictive Value (0.95 CI)	0.54 (0.53-0.56)	0.48 (0.46-0.49)	0.54 (0.53-0.56)	0.48 (0.46-0.49)
Genotype-Phenotype				
Condition +	2,124[1]	2,412[2]	2,124	2,412
CNV + / Condition +	116	123	84	88
CNV + / Condition -	63	56	22	18
CNV - / Condition -	2,360	2,079	2,401	2,117
CNV - / Condition +	2,008	2,289	2,040	2,324
Total Probands and Siblings	4,547	4,547	4,547	4,547

VUS = variant of unknown significance

* Genotype refers to the identification of ASD-relevant CNVs observed in infant siblings

1 ASD probands (n=2,124)

2 ASD probands (n=2,124) + non-ASD sibs with atypical development (n=288) (see "Simons Simplex Collection (SSC) to assess CNV false discovery" in Methods)

Supplementary Table 8: Broader Autism Phenotype Questionnaire (BAPQ) summary by family type

	**Simplex families (n = 157)**	**Multiplex families (n=86)**
**BAPQ subscale**	Average Score (SD)	Average Score (SD)
**Aloof**		
Mother	2.70 (0.9)	2.84 (1.0)
Father	3.05 (0.9)	3.09 (1.0)
**Pragmatic language**		
Mother	2.46 (0.7)	2.40 (0.8)
Father	2.28 (0.6)	2.56 (0.7)
**Rigidity**		
Mother	2.87 (0.6)	3.14 (0.9)
Father	3.00 (0.7)	2.99 (1.0)
**Total score**		
Mother	2.62 (0.6)	2.79 (0.8)
Father	2.84 (0.6)	2.88 (0.8)

Supplementary Note 1

Additional dataset acknowledgements

Funding support for the Study of Addiction: Genetics and Environment (SAGE) was provided through the NIH Genes, Environment and Health Initiative [GEI] (U01 HG004422). SAGE is one of the genome-wide association studies funded as part of the Gene Environment Association Studies (GENEVA) under GEI. Assistance with phenotype harmonization and genotype cleaning, as well as with general study coordination, was provided by the GENEVA Coordinating Center (U01 HG004446). Assistance with data cleaning was provided by the National Center for Biotechnology Information. Support for collection of datasets and samples was provided by the Collaborative Study on the Genetics of Alcoholism (COGA; U10 AA008401), the Collaborative Genetic Study of Nicotine Dependence (COGEND; P01 CA089392), and the Family Study of Cocaine Dependence (FSCD; R01 DA013423). Funding support for genotyping, which was performed at the Johns Hopkins University Center for Inherited Disease Research, was provided by the NIH GEI (U01HG004438), the National Institute on Alcohol Abuse and Alcoholism, the National Institute on Drug Abuse, and the NIH contract "High throughput genotyping for studying the genetic contributions to human disease" (HHSN268200782096C). The datasets used for the analyses described in this manuscript were obtained from dbGaP at http://www.ncbi.nlm.nih.gov/projects/gap/cgi-bin/study.cgi?study_id=phs000092.v1.p1 through dbGaP accession number phs000092.v1.p.

The authors acknowledge the contribution of data from Genetic Architecture of Smoking and Smoking Cessation accessed through dbGAP (https://www.ncbi.nlm.nih.gov/projects/gap/cgi-bin/study.cgi?study_id=phs000404.v1.p1, accession number phs000404.v1.p1). Funding support for genotyping, which was performed at

the Center for Inherited Disease Research (CIDR), was provided by 1 X01 HG005274-01. CIDR is fully funded through a federal contract from the National Institutes of Health to The Johns Hopkins University, contract number HHSN268200782096C. Assistance with genotype cleaning, as well as with general study coordination, was provided by the Gene Environment Association Studies (GENEVA) Coordinating Center (U01 HG004446). Funding support for collection of datasets and samples was provided by the Collaborative Genetic Study of Nicotine Dependence (COGEND; P01 CA089392) and the University of Wisconsin Transdisciplinary Tobacco Use Research Center (P50 DA019706, P50 CA084724).

The NEI Refractive Error Collaboration (NEIREC) Database found at https://www.ncbi.nlm.nih.gov/projects/gap/cgi-bin/study.cgi?study_id=phs000303.v1.p1 through dbGaP accession number phs000303.v1.p1 was also used for analyses. Funding support for NEIREC was provided by the National Eye Institute. We would like to thank NEIREC participants and the NEIREC Research Group for their valuable contribution to this research."

Funding support for the "CIDR Visceral Adiposity Study" was provided through the Division of Aging Biology and the Division of Geriatrics and Clinical Gerontology, NIA. The CIDR Visceral Adiposity Study includes a genome-wide association study funded as part of the Division of Aging Biology and the Division of Geriatrics and Clinical Gerontology, NIA. Assistance with phenotype harmonization and genotype cleaning, as well as with general study coordination, was provided by Heath ABC Study Investigators. This dataset was obtained from dbGaP at https://www.ncbi.nlm.nih.gov/projects/gap/cgi-bin/study.cgi?study_id=phs000169.v1.p1 through accession number phs000169.v1.p1.

We are grateful to all of the families at the participating Simons Simplex Collection (SSC) sites, as well as the principal investigators (A. Beaudet, R. Bernier, J. Constantino, E.

Cook, E. Fombonne, D. Geschwind, R. Goin-Kochel, E. Hanson, D. Grice, A. Klin, D. Ledbetter, C. Lord, C. Martin, D. Martin, R. Maxim, J. Miles, O. Ousley, K. Pelphrey, B. Peterson, J. Piggot, C. Saulnier, M. State, W. Stone, J. Sutcliffe, C. Walsh, Z. Warren, E. Wijsman).

Supplementary References

1. Yuen, R.K., et al. Genome-wide characteristics of de novo mutations in autism. NPJ Genom Med 1, 160271-1602710 (2016).
2. Pinto, D., et al. Functional impact of global rare copy number variation in autism spectrum disorders. Nature 466, 368-372 (2010).
3. Feuk, L., et al. Absence of a paternally inherited FOXP2 gene in developmental verbal dyspraxia. Am J Hum Genet 79, 965-972 (2006).
4. Yuen, R.K., et al. Whole genome sequencing resource identifies 18 new candidate genes for autism spectrum disorder. Nat Neurosci 20, 602-611 (2017).
