# Supplementary material for: Predictive impact of rare genomic copy number variations in siblings of individuals with autism spectrum disorders
Source: Nat Commun. 2019 Dec 5;10:5519. doi: 10.1038/s41467-019-13380-2 (PMC6892938; doi:10.1038/s41467-019-13380-2)
Supplement: Supplementary file 5 — Reporting Summary [file 41467_2019_13380_MOESM5_ESM.pdf]

nature research

Corresponding author(s): Stephen W. Scherer

Last updated by author(s): October 25, 2019

Reporting Summary

Nature Research wishes to improve the reproducibility of the work that we publish. This form provides structure for consistency and transparency in reporting. For further information on Nature Research policies, see Authors & Referees and the Editorial Policy Checklist .

Statistics

For all statistical analyses, confirm that the following items are present in the figure legend, table legend, main text, or Methods section.

n/a	Confirmed	
☐	☒	The exact sample size (*n*) for each experimental group/condition, given as a discrete number and unit of measurement
☒	☐	A statement on whether measurements were taken from distinct samples or whether the same sample was measured repeatedly
☒	☐	The statistical test(s) used AND whether they are one- or two-sided<br>*Only common tests should be described solely by name; describe more complex techniques in the Methods section.*
☒	☐	A description of all covariates tested
☒	☐	A description of any assumptions or corrections, such as tests of normality and adjustment for multiple comparisons
☐	☒	A full description of the statistical parameters including central tendency (e.g. means) or other basic estimates (e.g. regression coefficient) AND variation (e.g. standard deviation) or associated estimates of uncertainty (e.g. confidence intervals)
☒	☐	For null hypothesis testing, the test statistic (e.g. *F*, *t*, *r*) with confidence intervals, effect sizes, degrees of freedom and *P* value noted<br>*Give P values as exact values whenever suitable.*
☒	☐	For Bayesian analysis, information on the choice of priors and Markov chain Monte Carlo settings
☒	☐	For hierarchical and complex designs, identification of the appropriate level for tests and full reporting of outcomes
☒	☐	Estimates of effect sizes (e.g. Cohen's *d*, Pearson's *r*), indicating how they were calculated

Our web collection on statistics for biologists contains articles on many of the points above.

Software and code

Policy information about availability of computer code

Data collection	No software was used.
Data analysis	The following 6 algorithms were used, in different combinations across multiple platforms, to detect copy number variation: Chromosome Analysis Suite (ChAS) (version 3.0), Nexus (version 7), Partek Genomics Suite (version 6.4), iPattern, PennCNV and QuantiSNP. The package "epiR: Tools for the Analysis of Epidemiological Data" downloaded in R was used to tabulate all predictive value statistics and associated confidence intervals.

For manuscripts utilizing custom algorithms or software that are central to the research but not yet described in published literature, software must be made available to editors/reviewers. We strongly encourage code deposition in a community repository (e.g. GitHub). See the Nature Research guidelines for submitting code & software for further information.

Data

Policy information about availability of data

All manuscripts must include a data availability statement. This statement should provide the following information, where applicable:

- Accession codes, unique identifiers, or web links for publicly available datasets
- A list of figures that have associated raw data
- A description of any restrictions on data availability

All microarray data associated with the Infant Sibling Study can be accessed using the dbGaP accession ID phs001876.v1.p1. Related whole genome sequencing data is publicly available through the MSSNG database (www.mss.ng).

Field-specific reporting

Please select the one below that is the best fit for your research. If you are not sure, read the appropriate sections before making your selection.

- [x] Life sciences
- [ ] Behavioural & social sciences
- [ ] Ecological, evolutionary & environmental sciences

For a reference copy of the document with all sections, see nature.com/documents/nr-reporting-summary-flat.pdf

Life sciences study design

All studies must disclose on these points even when the disclosure is negative.

Sample size	Sample size was based on how many families from the Infant Siblings Study consented to be part of this ethics-approved study.
Data exclusions	No data was excluded from this analysis.
Replication	As previously stated the study could not be strictly replicated because there is no equivalent group of subjects with genotype data. At the request of reviewers, we did, however, replicate our analysis in a cohort of probands and unaffected siblings from the Simons Simplex Collection to assess the false-discovery rate of clinically-relevant copy number variants in non-ASD individuals. In doing so, we were also able to ascertain the frequency of clinically-relevant copy number variants in probands from the that same cohort and compare it to that obtained in our study.
Randomization	Individuals were included in the experimental group as they were recruited.
Blinding	Copy number variation analysis was performed while blinded to an individual's diagnostic status.

Reporting for specific materials, systems and methods

We require information from authors about some types of materials, experimental systems and methods used in many studies. Here, indicate whether each material, system or method listed is relevant to your study. If you are not sure if a list item applies to your research, read the appropriate section before selecting a response.

Materials & experimental systems

n/a	Involved in the study	
x		Antibodies
x		Eukaryotic cell lines
x		Palaeontology
x		Animals and other organisms
	x	Human research participants
x		Clinical data

Methods

n/a	Involved in the study	
x		ChIP-seq
x		Flow cytometry
x		MRI-based neuroimaging

Human research participants

Policy information about studies involving human research participants

Population characteristics	In order to qualify as a high-risk infant sibling, subjects had to have at least one older sibling (i.e. proband) with a diagnosis of autism spectrum disorder (ASD) and be less than 36 months of age. These infants were recruited and followed by expert clinicians until approximately 3 years of age, at which point their diagnostic status with respect to ASD was determined.
Recruitment	Participants were recruited as previously described in Ozonoff et al. (2011). These methods included recruiting individuals from clinics serving individuals with ASD, community events and using multi-media advertisements to increase awareness of study recruitment.
Ethics oversight	This study was approved by the Research Ethics Board (REB) at The Hospital for Sick Children (REB # 0019980189).

Note that full information on the approval of the study protocol must also be provided in the manuscript.
