# Supplementary material for: Predictive impact of rare genomic copy number variations in siblings of individuals with autism spectrum disorders
Source: Nat Commun. 2019 Dec 5;10:5519. doi: 10.1038/s41467-019-13380-2 (PMC6892938; doi:10.1038/s41467-019-13380-2)
Supplement: Supplementary file 6 — Description of Additional Supplementary Files [file 41467_2019_13380_MOESM6_ESM.pdf]

Title: Supplementary Data File 1.
Description: ASD-Relevant CNVs identified in probands and unaffected siblings from the Simons Simplex Collection 2.

Title: Supplementary Data File 2:
Description: List of rare CNVs 3.

Title: Supplementary Data File 3:
Description: List of ASD and neurodevelopmental delay candidate and risk genes
